# What Drives the Changing Characteristics of Plankton in Floodplain Lakes: Season, Environmental Factors, or Lake Characteristics?

**DOI:** 10.1002/ece3.74277

**Published:** 2026-09-09

**Authors:** Lingli Jiang, Zhongze Zhou, Bohan Zhou, Yaqiang Yuan, Su Mei, Yuqian Liu, Yutao Wang

**Affiliations:** ^1^ School of Resources and Environmental Engineering Anhui University Hefei China; ^2^ Anhui Shengjin Lake Wetland Ecology National Long‐Term Scientific Research Base Dongzhi China; ^3^ School of Chemistry and Chemical Engineering Anhui University Hefei China

**Keywords:** coupling effects, environmental variables, plankton, the Yangtze River

## Abstract

The floodplain lake ecosystem is one of the most biodiverse ecosystems on Earth. Its water level changes with seasonal floods. These periodic water level fluctuations have far‐reaching impacts on ecosystem structure and function. Systematic understanding of the structural characteristics of plankton communities in floodplain lakes, the long‐term coupled effects of environmental variables, and multi‐pathway action mechanisms will facilitate the accurate revelation of plankton adaptation strategies in dynamic hydrological environments. This study investigated the structure, taxonomic and functional diversity, community assembly, and niche differentiation of plankton communities in floodplain lakes in the middle and lower reaches of the Yangtze River during dry and flood seasons to identify key driving factors and coupled environmental effects. The results showed that plankton communities were consistently dominated by Cyanobacteria, Chlorophyta, Bacillariophyta, Cryptophyta, and Rotifera across four lake types, although their relative contributions varied markedly with hydrological season and lake type. Plankton density and taxonomic α‐diversity were significantly higher in the flood season, whereas both taxonomic and functional β‐diversity were lower, indicating enhanced community homogenization under high hydrological connectivity. Taxonomic β‐diversity was primarily driven by species turnover, while functional β‐diversity was dominated by nestedness, suggesting substantial functional redundancy among lakes. Neutral community models showed poor fit (*R* ^2^ < 0), and niche breadth analyses revealed significant seasonal contraction from flood to dry seasons, indicating that deterministic processes governed plankton community assembly. Environmental filtering associated with water temperature, water depth, turbidity, and nutrient availability, together with niche differentiation, played dominant roles in shaping plankton communities. Structural equation modeling further demonstrated that land use and lake type indirectly regulated plankton density and diversity through their coupled effects on water quality and nutrient conditions, with hydrological seasonality exerting the strongest overall influence. These findings provide a scientific basis for ecological assessment and management of floodplain lakes.

## Graphical abstract

In this study, we conducted investigations and monitoring on lakes during the wet season and dry season. We studied the structure, taxonomic/functional diversity, community assembly, and niche differentiation of plankton communities, analyzed the biogeographical patterns of plankton communities, and explored the driving factors affecting plankton communities as well as the coupling effects of environmental variables. This study provides basic data for understanding the characteristics of plankton communities in subtropical floodplain lakes and also offers a scientific basis for the management and protection of floodplain lake ecosystems.

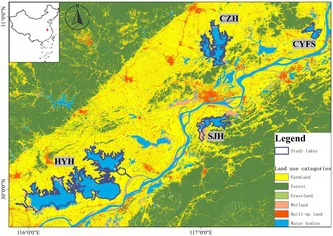

## 1 Introduction

Floodplains are primarily formed by the periodic flooding of rivers (Gordon et al. 2020; Rajib et al. 2023). When the water levels of rivers rise, floods inundate the surrounding areas with lower terrain. The long‐term and recurring flood encroachments have gradually led to the formation of lakes in these submerged areas. Floodplain lakes are important components of river–floodplain systems and provide diverse ecological functions and ecosystem services. They play a key role not only in flood regulation, maintenance of biodiversity, and promotion of material cycling, but also provide humans with abundant water resources and fishery resources (Li et al. 2019; Lubinski et al. 2008; Zeng et al. 2024). Water level changes of floodplain lakes are closely coupled with seasonal and interannual fluctuations of the main river: during flood seasons, rising main river levels cause water to overflow into floodplain lakes and elevate their levels; during dry seasons, receding main river levels lead to lake water flowing back to the main river, and this linkage further affects adjacent connected small wetland water bodies (Nabout et al. 2006; Zhang, Cai, et al. 2024; Zhang, Liu, and Chen 2024). Floodplains are widely distributed across many regions worldwide (the Amazon Basin, the middle‐lower reaches of the Yangtze River, the Rhine River Basin in Europe), and such significant water level fluctuations not only shape their unique wetland ecosystems but also require the organisms in the lakes to have strong adaptive capabilities (Kumari et al. 2024; Zhao et al. 2024). For example, submerged plants elongate their stems during high‐water periods to obtain sufficient light (Xie et al. 2023); fish actively move into floodplain shallows to spawn and forage during flood seasons, thereby adapting to periodic water level fluctuations.

Plankton are a core component of the floodplain lake ecosystem and serve as an important indicator for assessing lake ecological functions (material cycling, water quality regulation, and biodiversity conservation) and maintaining the structural integrity of lakes to resist environmental changes. Environmental factors such as nutrient concentration in the water, land use patterns, lake type, seasonal changes, and periodic hydrological fluctuations all affect the survival and distribution of plankton. Studies have shown that nutrient concentration in the water directly affects the density of phytoplankton, thereby indirectly controlling the distribution of zooplankton (Li et al. 2020). Due to differences in hydrological characteristics and ecological conditions, different types of lakes form phytoplankton communities with distinct characteristics (Zhang, Cai, et al. 2024; Zhang, Liu, and Chen 2024). Changes in land use patterns may alter nutrient inputs and the physicochemical properties of the water, thereby affecting the structure of plankton communities (Lv et al. 2024). Additionally, seasonal variations in environmental factors such as water temperature, light conditions, and precipitation play a key role in the growth, metabolism, and reproduction of plankton (Tan et al. 2009). Periodic hydrological changes in floodplain lakes often alter the plankton's living environment and impact the biodiversity of communities (Wang et al. 2020). According to the intermediate disturbance hypothesis, community diversity typically reaches its highest level under moderate disturbance (Connell 1978). Frequent hydrological disturbances in floodplain lakes help maintain high species richness and evenness while environmental filtering selects functional trait groups that are better adapted, thus maintaining the diversity of functional traits in the community (Guo et al. 2021; Zhou et al. 2024). The driving effects of nutrients, land use, seasonal changes, and hydrological fluctuations on plankton may not operate independently. Instead, they may produce additive, synergistic, or antagonistic effects on plankton communities by altering nutrient supply, habitat conditions, hydrological connectivity, and environmental filtering processes. Moreover, the dominant drivers of plankton community variation may differ among lake types, seasons, and hydrological conditions, leading to inconsistent conclusions regarding their combined effects.

One of the core objectives of ecological research is to reveal the spatiotemporal differences and assembly processes of biological communities. Geographical distance and environmental distance are key factors shaping the compositional differences of plankton communities. By comparing β‐diversity and distance decay effects among communities, the spatiotemporal variation patterns of the study area can be revealed (Soininen et al. 2007). β‐diversity reveals the differences in species composition between different communities and can be decomposed into turnover components (species replacement between locations) and nested components (one community's species set is a subset of another community) (Baselga 2010). The former is often associated with species replacement driven by environmental filtering, whereas the latter is more commonly related to dispersal limitation. Understanding the dominance of these components can provide more precise scientific guidance for water resource management and biodiversity conservation in floodplain lakes. Community assembly is key to understanding the structure, function, and dynamics of plankton communities. Deterministic processes (such as environmental filtering and niche differentiation) and stochastic processes (such as species dispersal and random population fluctuations, including birth, death, and reproduction) are complementary mechanisms that shape plankton communities (Mo et al. 2021; Zhang, Xu, et al. 2022; Zhang, Yang, et al. 2022). Differences in the environmental adaptability of plankton drive niche differentiation, promote species coexistence, and enhance community stability (Hampton 2005; Lengyel et al. 2020). Increased hydrological connectivity facilitates plankton dispersal, which may reduce dispersal limitation and enhance the role of stochastic processes in structuring communities (Zhang, Xu, et al. 2022; Zhang, Yang, et al. 2022).

This study focused on floodplain lakes in the middle and lower reaches of the Yangtze River, investigating plankton community structure, taxonomic and functional diversity, community assembly, and niche differentiation between dry and flood seasons. It aimed to clarify how hydrological seasonality, environmental conditions, and lake characteristics jointly shape plankton community patterns in subtropical floodplain lakes. Based on this goal, we hypothesized that (1) plankton communities exhibit higher density and α‐diversity but lower β‐diversity during the flood season compared with the dry season; (2) plankton community assembly is predominantly governed by deterministic processes driven by environmental filtering and niche differentiation; (3) phytoplankton and zooplankton display distinct biogeographical patterns and different seasonal response modes; (4) changes in plankton characteristics are jointly driven by multiple factors, including hydrological seasonality, environmental conditions, and lake characteristics. Through this study, we expect to enhance the understanding of plankton community characteristics in subtropical floodplain lakes and provide a scientific basis for the management and protection of floodplain lake ecosystems.

## 2 Materials and Methods

### 2.1 Study Sites and Sampling Campaign

The studied lakes (116°00′ E–117°43′ E, 29°52′ N–31°16′ N) are on the north or south bank of the Yangtze River in Anhui Province, China. The climate is humid and subtropical with significant seasonal changes that are affected by the monsoons. The largest amount of rain falls from May to August (Zhang et al. 2019). These lakes connected to the Yangtze River maintain a high degree of hydraulic connectivity with the river. During periods of elevated Yangtze River water levels, the riverine flow inundates the lakes; conversely, when the river's water level recedes, the lakes serve to recharge the Yangtze River, thereby establishing an integrated river–lake ecosystem.

Seven lakes within the floodplain were selected and further classified into four geographic regions, collectively referred to as lakes hereinafter. Their water‐level fluctuation patterns and hydrological characteristics are described as follows. Shengjin Lake (SJH) exhibits water‐level fluctuations similar to those of the Yangtze River mainstream because its control sluices remain permanently open, maintaining continuous hydrological connectivity with the Yangtze River system. Water levels decline during winter and spring, resulting in partial seasonal desiccation of the lake. Huayanghe Lakes (HYH) comprise a complex water system consisting of Longgan Lake, Huangda Lake, and Bo Lake, which together form a fully connected hydrological unit. Unlike Shengjin Lake, water levels in this lake group are maintained at a relatively stable level through artificial regulation of the river‐connected sluices (operating cycle: open from June to September and closed from October to May), resulting in transitional hydrological characteristics between natural and artificially regulated systems. Caizi Lake (CZH) is an important component of the Yangtze‐to‐Huaihe Water Diversion Project. During non‐diversion periods, it is operated under strict engineering regulations, with the sluices remaining closed for more than 95% of the time, thereby forming a highly regulated hydrological system that differs markedly from those of natural lakes. Water levels in Chenyao Lake and Fengsha Lake (CYFS) remain stable throughout the year under dam regulation. Although a 12.5‐m‐high separation dam prevents water exchange between the two lakes, coordinated dam operation maintains similar hydrological characteristics in both lakes.

**FIGURE 1:**
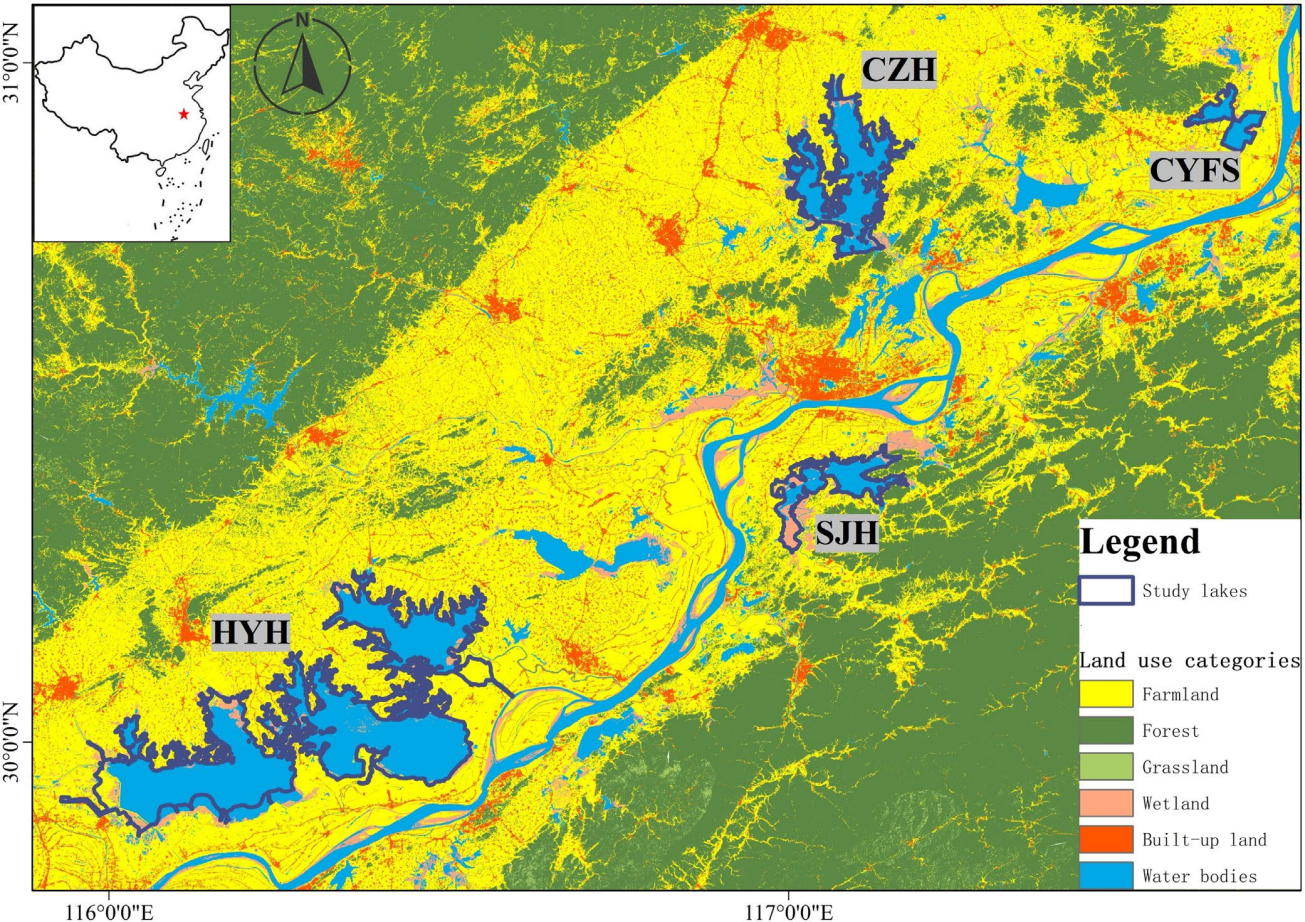
Map of the study area and sampling sections. Shengjin Lake (SJH), Huayanghe Lakes (HYH), Caizi Lake (CZH), Chenyao Lake and Fengsha Lake (CYFS).

In order to investigate the characteristics of plankton communities in floodplain lakes, we collected four times between 2021 and 2023 during the flood (July) and dry (December) seasons, namely, July 2021 in the CYFS (10 sampling sites), December 2021 in the CYFS (10 sampling sites), July 2023 in the SJH (11 sampling sites), HYH (15 sampling sites), and CZH (15 sampling sites), and December 2023 in the SJH (8 sampling sites), HYH (19 sampling sites), and CZH (16 sampling sites). A total of 104 samples were collected in this study. The reported numbers refer to the total sampling sites per lake type per season, rather than per individual lake. No extreme weather events occurred during sampling (Figure 1, Figure S1).

### 2.2 Data Collection

#### 2.2.1 Sampling and Laboratory Analysis

Water samples were taken in the seven lakes from July 2021 to December 2023. We measured water temperature (WT), dissolved oxygen (DO), and electrical conductivity (EC) in situ using the Hach HQ40d portable multimeter, and turbidity (Turb) with the Hach 2100Q. Secchi depth (SD) was measured in situ using a Secchi disk, while water depth (WD) was measured using a portable depth sounder. At each sampling point, we collected water samples in polypropylene plastic bottles and brought them to the laboratory within 48 h to determine the nutrient concentrations, including ammonia (${\mathrm{NH}}_{4}^{+}$‐N) (HJ 535‐2009), total nitrogen (TN) (HJ 636‐2012), total phosphorus (TP) (GB 11893‐1989), and chlorophyll‐a (Chla) (HJ 897‐2017).

The average depth of the lakes surveyed in this study is less than 5 m. Owing to their shallow bathymetry, surface water samples are considered representative of the phytoplankton community throughout the entire water column. Accordingly, only surface water was collected for analysis. Phytoplankton and Rotifera were collected in 1‐L plastic bottles and then fixed with Lugol's iodine solution. The concentrated samples were allowed to settle for 48 h and then concentrated to 30 mL. Samples used for assessing zooplankton (Copepoda, Cladocera) densities were collected by filtering 20 L of water through a 64‐μm mesh from the different sampling sites of the lakes, and then concentrated into 50 mL plastic bottles and immediately preserved in the field in a 4% formaldehyde solution. Plankton individuals at the lowest allowable taxonomic level were enumerated and identified according to the Chinese national standard (GBT12763.6‐2007) microscopy method, and each identified taxonomic name of the plankton was validated against the classification system proposed by Sun et al. (1999) and Zhou et al. (2020).

#### 2.2.2 Land Use Data

Utilizing a digital elevation model (DEM) with a resolution of 1 km, we delineated the watershed boundaries of the four study lakes. Subsequently, land use types within a 5‐km buffer zone around the watershed boundary of the study lakes in 2023 were visually interpreted and extracted from 30 m resolution Landsat 9 OLI‐2/TIRS‐2 images acquired on August 6, 2023 utilizing ArcGIS 10.8.1 software (Esri, Redlands, CA). Land types were distinguished based on spectral signatures, texture, shape and spatial distribution characteristics, and field sampling was implemented to validate classification results. The land use categories were refined into six distinct classifications: (1) farmland (including paddy fields and dry land), (2) forest, (3) grassland, (4) wetland, (5) built‐up land (including urban areas, rural residential areas, and industrial zones), and (6) water bodies (including rivers, lakes, reservoirs, and ponds) (Zhang 2021).

#### 2.2.3 Trait Information

To ascertain the functional classification of zooplankton species, we used seven functional traits: size, habitat, feeding type, trophic group, life span, reproduction, and predatory escape response (Table 1, Table S1). By studying six functional traits: size, nitrogen‐fixation, mobility, morphology, cell‐wall, and metabolism, we established the functional classification of phytoplankton species (Table 2, Table S2). We selected these traits because they describe the responses of the organisms to environmental conditions and their impacts on ecosystem processes (Barnett et al. 2007; Edwards et al. 2015; Litchman and Klausmeier 2008).

**TABLE 1:** Morphological, physiological, and behavioral traits of the zooplankton species that were considered in the analysis.

Traits	Type	Values	Ecological function
Size	Morphological	Rotifer; small (< 500 μm); middle (500–1000 μm); large (> 1000 μm)	Reproduction; predation; predator avoidance
Habitat	Behavioral	Planktonic; benthic	Predation; food web status; predator avoidance
Trophic group	Physiological	Omnivorous; herbivore	Predation; food web status
Feeding type	Behavioral	Sugador‐R; filtration‐R; predator‐R; raptorial‐Cop; scraper‐Clad; filtration‐Clad	Predation; food web status
Life span	Physiological	Long; short	Reproduction
Reproduction	Physiological	Asexual; sexual	Reproduction
Predatory escape response	Behavioral	Low; medium; big	Predation; predator avoidance

*Note:* A list of species with each trait can be found in Table S1, accompanied by the corresponding references.

**TABLE 2:** Morphological, physiological, and behavioral traits of the phytoplankton species that were considered in the analysis.

Traits	Type	Values	Ecological function
Size	Morphological	Low (< 100 μm^3^); medium (100–1000 μm^3^); high (> 1000 μm^3^)	Reproduction; resource acquisition; predator avoidance
Nitrogen‐fixation	Physiological	No‐fixation; heterocysts; fixation without heterocysts	Resource acquisition
Mobility	Behavioral	Sessile; oscillation; aerotopes; flagellum; raphe movement	Resource acquisition; predator avoidance
Morphology	Morphological	Colony; filament; unicell; coenobium	Resource acquisition; predator avoidance
Cell‐wall	Morphological	Mucilage; murein; cellulose; pellicle; lorica; silicified	Predator avoidance
Metabolism	Physiological	Autotrophic; mixotrophic	Resource acquisition

*Note:* Note: A list of species with each trait can be found in Table S2, accompanied by the corresponding references.

### 2.3 Data Analyses

All data analyses were performed in R (version 4.3.1) and the results were visualized by the “*ggplot2*” package.

We chose Shannon–Wiener's index, Pielou's evenness index, and Margalef's index to characterize the taxonomic alpha diversity of phytoplankton and zooplankton (*vegan* package). Functional richness index (FRic), functional evenness index (FEve), and functional dispersion index (FDis) were utilized to describe the functional diversity (*mFD* package). Margalef's index and functional richness index, respectively, reflect the breadth of diversity from the taxonomic and functional perspectives. Pielou's index and functional evenness index, respectively, reflect the uniformity of diversity from the taxonomic and functional perspectives. Shannon–Wiener's index integrates species richness and evenness, while the functional dispersion index integrates trait dispersion and relative species abundance; both capture the overall level of community diversity. Alpha diversity differences between seasons were tested via Bonferroni‐corrected Dunn's test (*dunn. test* package).

Within the study lakes (SJH, HYH, CZH, and CYFS) and hydrological periods, taxonomic and functional β‐diversity was calculated among sampling sites. The multisite dissimilarity Sørensen index was calculated using the *betapart* package, and the total Sørensen dissimilarity was decomposed into spatial turnover (*β*_sim_) and nestedness (*β*_sne_) components employing the *agricolae* and *adespatial* packages. To explore the potential factors driving the variability of the plankton community, we analyzed the spatiotemporal community datasets. Such analysis is crucial for studying the spatiotemporal patterns of biodiversity and the mechanisms of diversity maintenance. The distance–decay relationship is a prominent community analytical framework describing how community similarity (or conversely, dissimilarity) changes with increasing geographical or environmental distance, reflecting the combined effects of dispersal limitation and environmental filtering. Bray–Curtis dissimilarity between samples was calculated using the *vegan* package and subsequently converted to community similarity (1 − dissimilarity) for regression analyses. Geographical distances between sampling sites were calculated using the geosphere package, and environmental distances were calculated as Euclidean distances based on standardized environmental variables using the *vegan* package. Ordinary least‐squares simple linear regression models were fitted using the lm function in the *stats* package, with community similarity as the response variable and geographical or environmental distance as the explanatory variable. Regression analyses were performed using functions implemented in the *stats* package in R, and the fitted relationships were visualized using the *ggplot2* package.

Non‐metric Multidimensional Scaling (NMDS) based on Euclidean distance was used to characterize environmental heterogeneity, while NMDS based on Bray‐Curtis distance can visualize compositional differences in plankton community structure (*vegan* package). Similarity analysis based separately on species‐level zooplankton and phytoplankton abundance (ANOSIM) was performed to examine the differences in plankton communities between periods (*vegan* package).

Community assembly was considered a result of multiple intertwined processes, including deterministic (niche‐based) and stochastic (neutrality‐based) mechanisms. The neutral community model of Sloan et al. (2006) was applied to plankton abundance data to assess the role of stochastic processes in community assembly. Species' mean relative abundance in the metacommunity and their occurrence frequency across samples were used to fit the model, with the immigration rate (*m*) estimated using nonlinear least squares. No permutation or randomization of samples, species identities, or abundances was performed; stochasticity was represented explicitly by the neutral model framework (*mobsim* package). The niche width of each phytoplankton or zooplankton species was calculated using Levin's niche breadth index via the *MicroNiche* package. Generalized linear models (GLMs) with a Gamma distribution and a log link function were used to assess differences between the flood and dry seasons in plankton niche breadth. This distribution was appropriate because niche breadth values (Levin's index) were continuous, strictly positive, and right‐skewed (*stats* package).

To reduce multicollinearity among the six land‐use variables and derive an integrated measure of land‐use composition, principal component analysis (PCA) was performed on the standardized proportional areas of farmland, forest, grassland, wetland, built‐up land, and water bodies. The scores of the first principal component (PC1), representing the dominant gradient in land‐use composition, were subsequently included as a continuous composite variable in the Spearman's rank correlation analysis and Mantel analyses.

Mantel analyses were primarily used to identify overall associations between environmental factors and plankton community structure (*linkET* package).

The environmental variables in this study were grouped into nutrients, water quality parameters, season, and lake characteristics. Nutrients included ammonia nitrogen (${\mathrm{NH}}_{4}^{+}$‐N), total nitrogen (TN), and total phosphorus (TP). Water quality parameters included dissolved oxygen (DO), electrical conductivity (EC), turbidity (Turb), and Secchi depth (SD). Season was represented by water temperature (WT) and water depth (WD), while lake characteristics included lake type and the proportions of six land‐use categories: farmland, forest, grassland, wetland, built‐up land, and water bodies. To examine the relationships between environmental factors and plankton relative abundance patterns of major taxonomic groups, Spearman's rank correlation analysis was conducted. For phytoplankton and zooplankton, community attributes were represented by the relative abundance of major taxonomic groups at the phylum (phytoplankton) or class (zooplankton) level, calculated based on individual density data. Relative abundance was used to reduce the influence of large differences in absolute abundance among sampling sites. All environmental variables were log(*x* + 1)‐transformed prior to analysis to reduce data skewness and the influence of extreme values. The correlation analysis was performed using the *stats* package.

Partial Least Squares Structural Equation Modeling (PLS‐SEM; *plspm* package) was used to evaluate the direct and indirect effects of land use, lake type, nutrients, water quality, and seasonal forcing on plankton attributes. Nutrients and water quality were treated as separate constructs to distinguish nutrient availability from other physicochemical conditions. Season was included as an exogenous latent variable represented by WT and WD rather than as a categorical variable. Higher values of the Season construct were interpreted as representing warmer and deeper conditions, which generally characterized the flood season. Two a priori models were constructed: one addressing plankton density and trophic interactions (nutrients–phytoplankton–zooplankton) and the other focusing on community diversity and functional diversity (*plspm* package). The path directions were specified a priori based on ecological hypotheses and were interpreted as hypothesized dominant relationships rather than definitive evidence of strictly unidirectional causality.

Spatial autocorrelation was tested to assess whether spatial dependence could bias statistical inference, as some sampling sites were geographically proximate. Moran's *I* was calculated for key ecological response variables, including phytoplankton and zooplankton diversity indices (Shannon–Wiener index, Pielou's evenness, Margalef's index, FRic, FEve, and FDis) as well as phytoplankton and zooplankton abundance. Spatial weights were constructed using inverse geographical distance based on sampling coordinates, with zero distances corrected using the minimum non‐zero distance. Significance was evaluated using permutation tests (*spdep* package). Moran's *I* therefore represented the combined spatial structure of within‐lake and among‐lake relationships, while shared limnological conditions were partly represented by the measured environmental variables and lake type included in subsequent analyses.

## 3 Results

### 3.1 Spatiotemporal Patterns of Environmental Variables

Table S3 summarized the measured physical and chemical factors of the study lakes. The environmental conditions of the sampling regions showed pronounced spatial and seasonal variations. In summer, water temperatures were relatively high across all lakes (28.08°C–31.85°C), while dissolved oxygen was comparatively low and nutrient levels were generally higher than in winter. Among the sites, CYFS exhibited the highest concentrations of total nitrogen, ammonia, and total phosphorus, indicating the most pronounced eutrophication, whereas HYH was characterized by low turbidity and high Secchi depth, reflecting relatively clear water conditions. SJH and CZH showed moderate to high nutrient and chlorophyll‐a concentrations, suggesting relatively high primary productivity. In winter, water temperatures decreased markedly (7.08°C–11.81°C), while dissolved oxygen generally increased, nutrient concentrations generally declined, and Secchi depth generally increased. However, these seasonal trends varied among lake types; notably, turbidity increased markedly in HYH during winter, in contrast to the other lake types. Thus, both environmental conditions and their temporal changes differed among lake types, reflecting the combined influences of seasonal variation and lake‐specific characteristics (Table S3).

**FIGURE 2:**
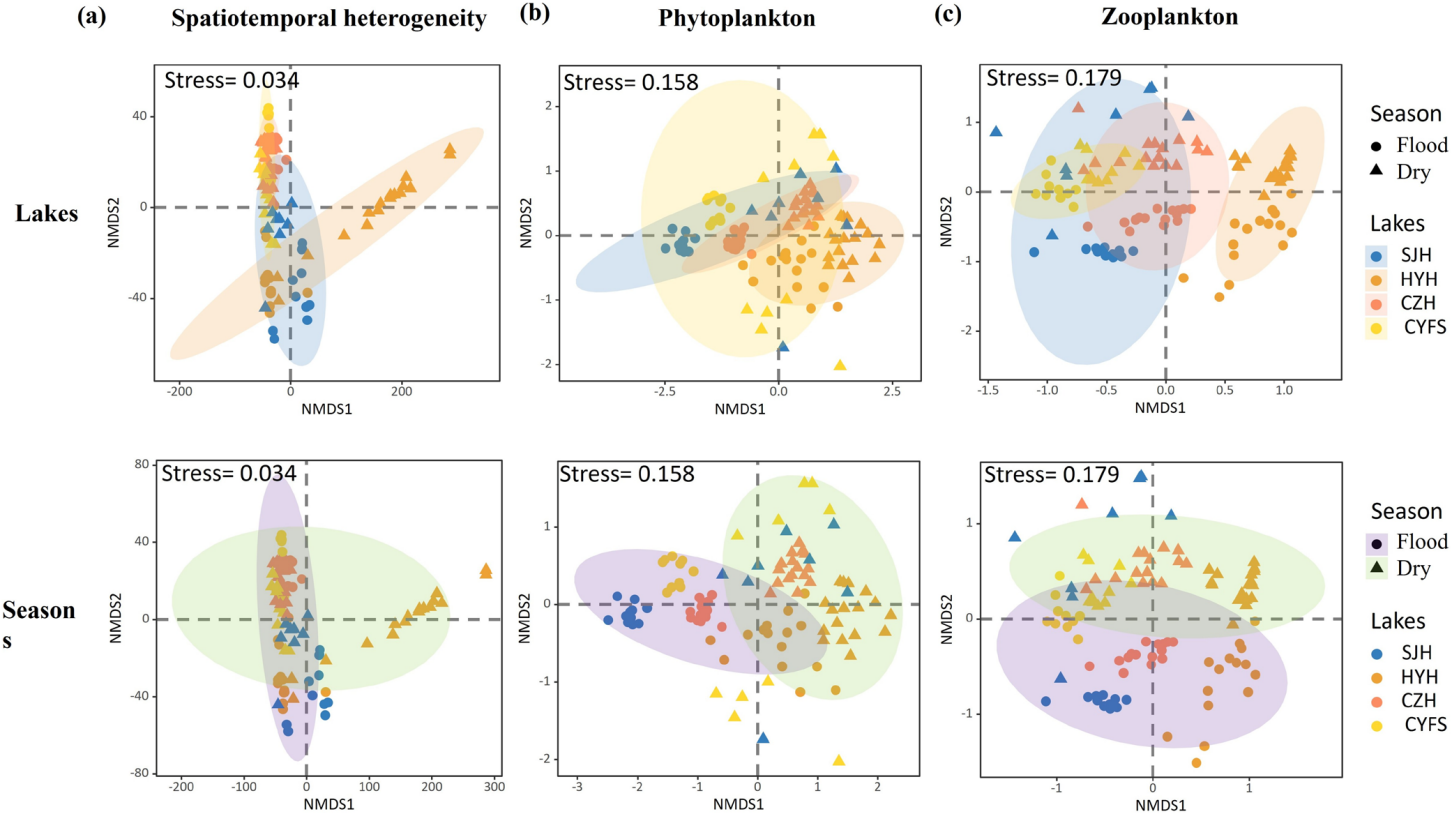
Non‐metric multidimensional scaling (NMDS) plots of the spatiotemporal heterogeneity of environmental variables and the composition of phytoplankton and zooplankton during sampling seasons. For each column, the upper and lower panels represent the same NMDS ordination, with ellipses grouped by lakes and seasons, respectively. (a) NMDS plots of the spatiotemporal heterogeneity of environmental variables based on Euclidean distances. (b) NMDS plots of the composition of phytoplankton based on Bray Curtis distances. (c) NMDS plots of the composition of zooplankton based on Bray Curtis distances.

The Non‐metric Multidimensional Scaling (NMDS) plot showed the spatiotemporal heterogeneity of environmental variables based on Euclidean distances. The results of the NMDS analysis from this study revealed the spatiotemporal heterogeneity characteristics of environmental variables among different seasons and lakes (Figure 2a). The stress values of the NMDS ordinations for environmental variables, phytoplankton, and zooplankton were 0.034, 0.158, and 0.179, respectively, all below 0.2, indicating reliable ordination fits.

The NMDS ordination showed partial separation between samples from the flood and dry seasons, although substantial overlap was observed, indicating some seasonal differences in overall environmental conditions (Figure 2a). Samples from the dry season exhibited a wider dispersion in ordination space than those from the flood season, suggesting greater environmental heterogeneity during the dry period. In addition, sampling sites differed in their spatial distribution patterns. Samples from CYFS and CZH formed relatively compact clusters, indicating similar environmental characteristics, whereas samples from HYH and SJH were more widely dispersed, highlighting stronger spatial heterogeneity among these lakes.

The NMDS ordination based on Bray–Curtis distances, supported by ANOSIM, showed significant differences in phytoplankton (*R* = 0.5173, *p* = 0.001) and zooplankton (*R* = 0.3189, *p* = 0.001) community composition among the four lake systems and hydrological periods (Figure 2b,c). The higher ANOSIM R value for phytoplankton indicated stronger overall group separation than for zooplankton.

### 3.2 Diversity and Composition of the Plankton Communities

**FIGURE 3:**
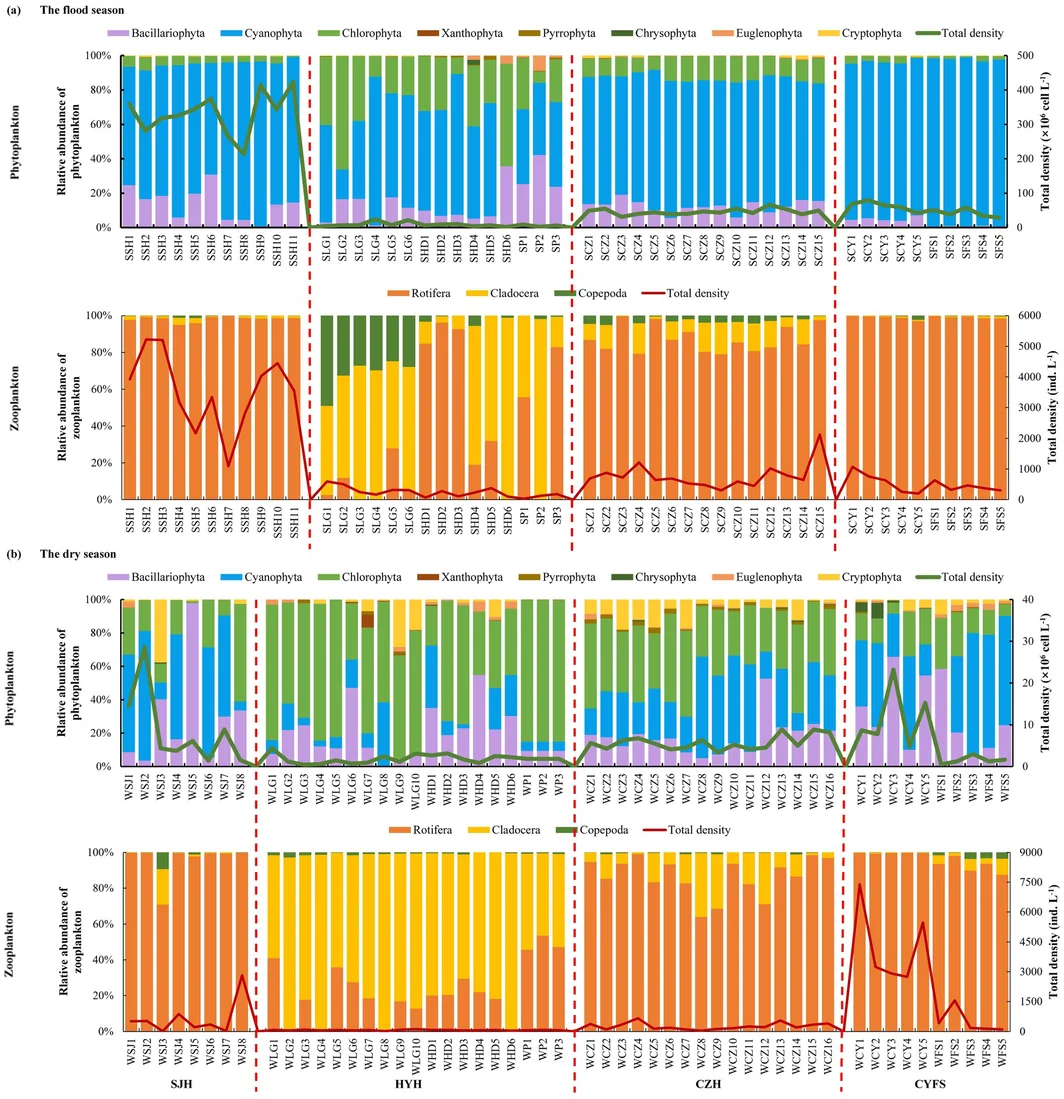
Variation in plankton taxa during each sampling season. (a) Variation in the relative abundance of different plankton taxa and total plankton density during the flood season. (b) Variation in the relative abundance of different plankton taxa and total plankton density during the dry season.

A total of 186 phytoplankton species and 62 zooplankton species were identified across the study lakes. Phytoplankton communities were mainly composed of Cyanobacteria, Chlorophyta, Bacillariophyta, and Cryptophyta. The relative dominance of these groups varied primarily between seasons, whereas differences among the study lakes were more evident in total phytoplankton density (Figure 3; Figure S2a; Table S5). Cyanobacteria showed high relative abundance across all study lakes and generally dominated during the flood season, particularly in CYFS. During the dry season, cyanobacterial dominance weakened, and the contribution of Chlorophyta increased across all study lakes, while Bacillariophyta and Cryptophyta also increased at some sampling sites. Total phytoplankton density varied markedly among lakes and sampling sites, with particularly high values in SJH during the flood season. At the species level, *Ulnaria acus*, *Stephanocyclus meneghinianus*, *Chroococcus dispersus* , and *Phormidium* sp. were widely distributed across lakes and seasons. Representative Chlorophyta included *Chlorella vulgaris* , *Desmodesmus communis*, and *Monoraphidium contortum* , while *Cryptomonas ovata* and *Komma caudata* were relatively abundant in several dry season samples.

Among the three zooplankton groups analyzed, Rotifera dominated in SJH, CZH, and CYFS during both seasons, whereas Cladocera became dominant in HYH during the dry season (Figure 3; Figure S2b). Zooplankton density also varied markedly among lakes and seasons. Representative taxa included *Asplanchna girodi* and *Trichocerca pusilla* (Rotifera), *Bosmina longirostris* (Cladocera), and *Cyclops vicinus* (Copepoda).

Moran's *I* analyses indicated weak to moderate spatial autocorrelation in several plankton diversity indices. For phytoplankton, Shannon–Wiener index, Pielou's evenness, Margalef's index, FRic, FEve, and FDis showed either weak but significant or non‐significant spatial autocorrelation (Moran's *I* ≤ 0.18). For zooplankton, the Shannon–Wiener index, Pielou's evenness, Margalef's index, and FRic showed significant spatial autocorrelation (Moran's *I* = 0.15–0.25, *p* < 0.001), whereas FEve and FDis showed no significant spatial autocorrelation (*p* > 0.05). Phytoplankton abundance showed no significant spatial autocorrelation (Moran's *I* = 0.028, *p* = 0.127), whereas zooplankton abundance exhibited weak but significant spatial autocorrelation (Moran's *I* = 0.12, *p* < 0.001). Overall, Moran's *I* values were below 0.3, indicating limited spatial dependence.

**FIGURE 4:**
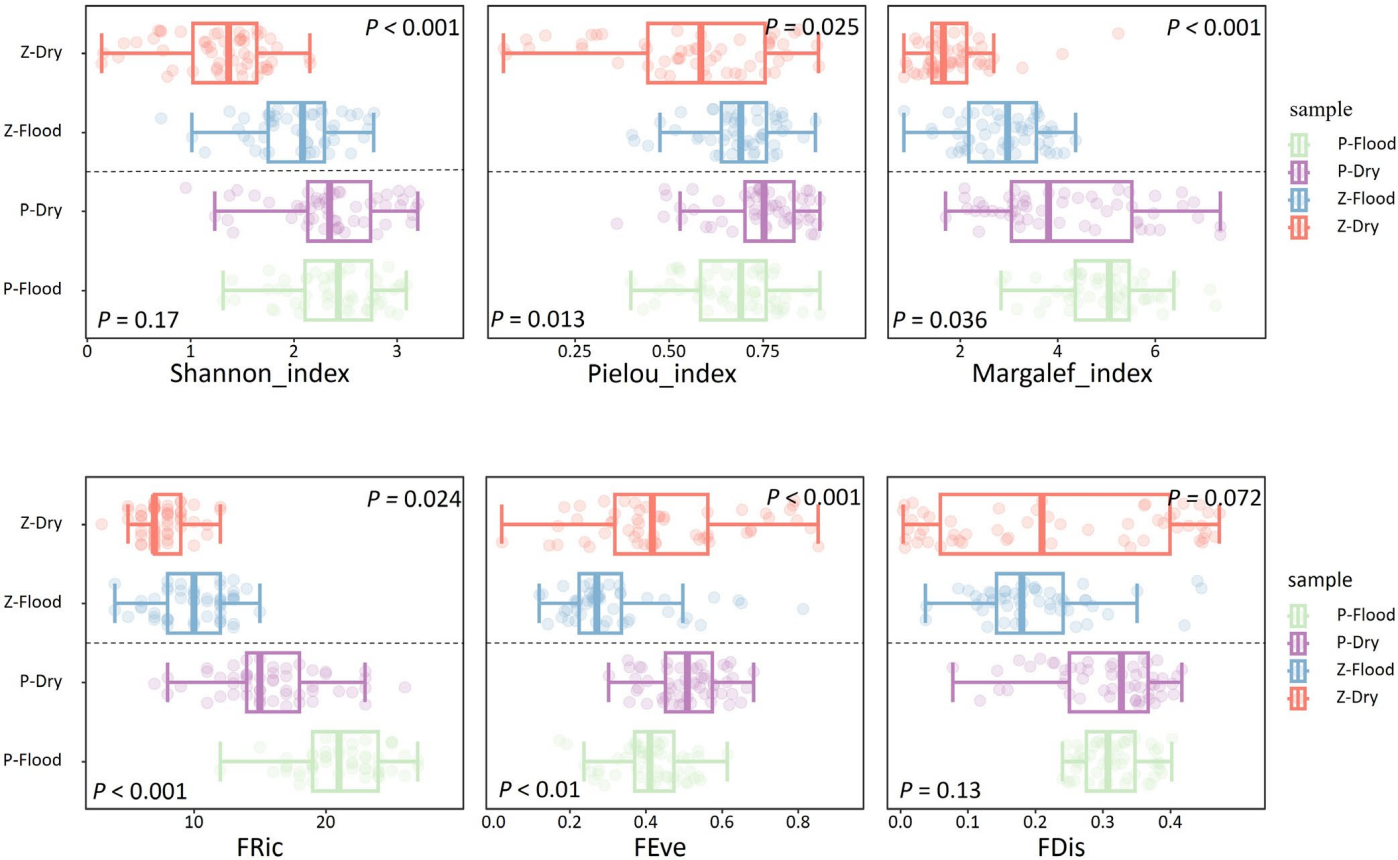
Box plot of the α diversity of plankton. Phytoplankton (P), Zooplankton (Z), Functional richness index (Fric), Functional evenness index (FEve), Functional dispersion index (FDis).

At the overall scale, samples from all four study lake systems were combined to compare plankton α‐diversity between the flood and dry seasons (Figure 4). For phytoplankton, Margalef's index (*p* = 0.036) and functional richness (*p* < 0.001) were higher in the flood season than in the dry season, whereas Pielou's evenness (*p* = 0.013) and functional evenness (*p* < 0.01) were higher in the dry season. No significant differences were detected in the Shannon–Wiener index (*p* = 0.17) or functional dispersion (*p* = 0.13) between the two hydrological periods. For zooplankton, the Shannon–Wiener index (*p* < 0.001), Pielou's evenness (*p* = 0.025), Margalef's index (*p* < 0.001), and functional richness (*p* = 0.024) were higher in the flood season, whereas functional evenness was higher in the dry season (*p* < 0.001). Functional dispersion did not differ significantly between the two hydrological periods (*p* = 0.072). Lake‐specific means and standard deviations of the diversity indices are provided in Table S4.

**FIGURE 5:**
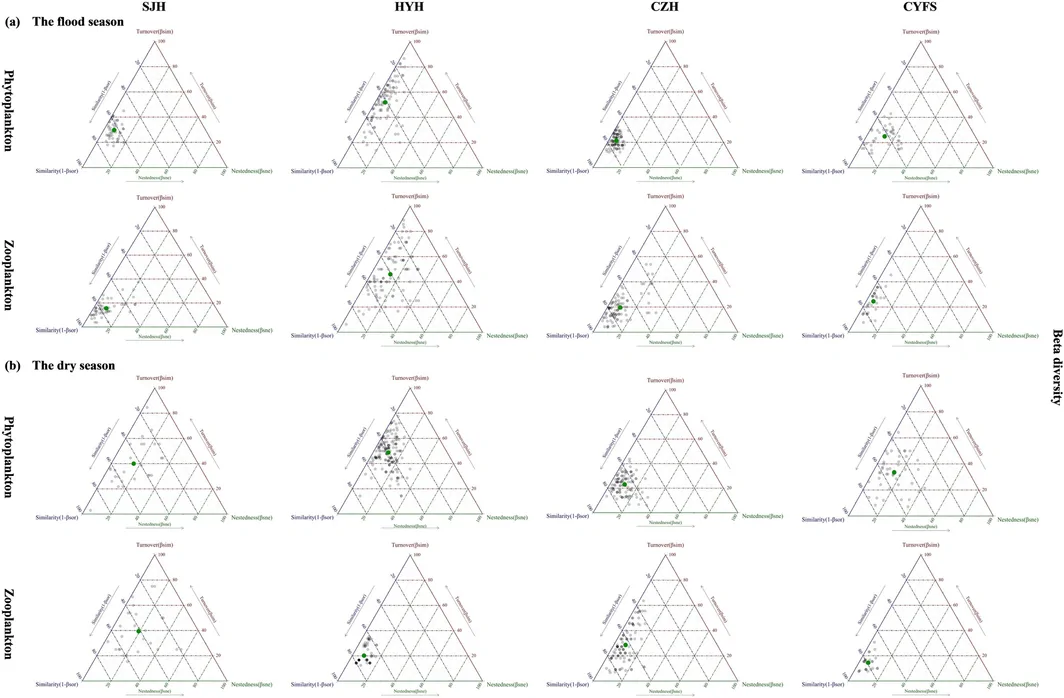
Ternary plot of the taxonomic β diversity of plankton. (a) Taxonomic β diversity of phytoplankton. (b) Taxonomic β diversity of zooplankton.

**FIGURE 6:**
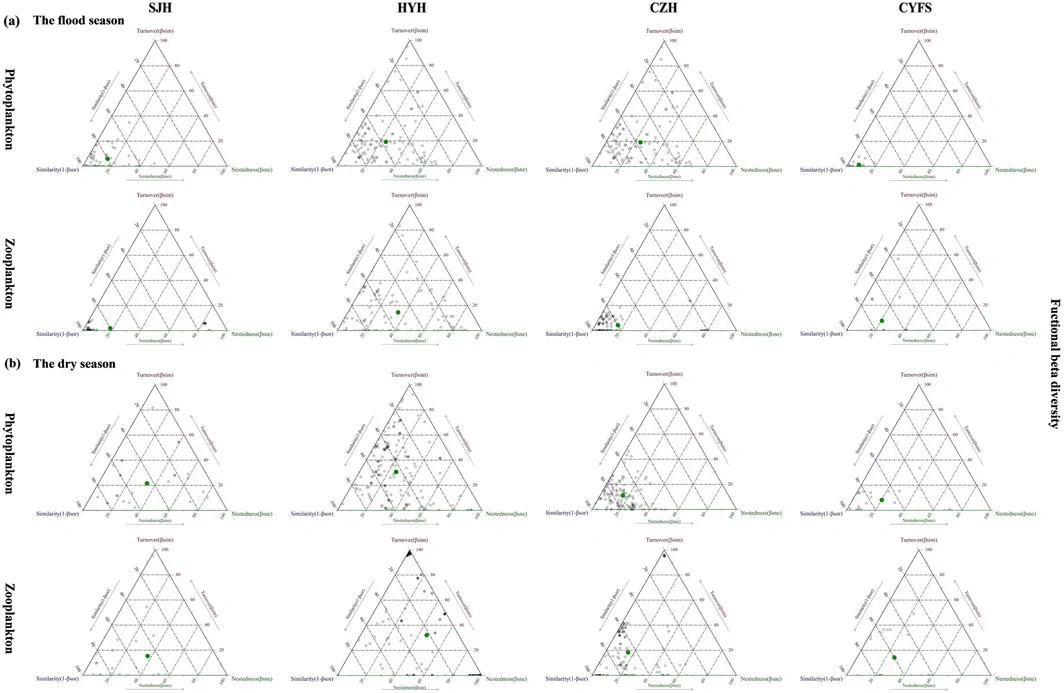
Ternary plot of the Functional β diversity of plankton. (a) Functional beta diversity of phytoplankton. (b) Functional beta diversity of zooplankton.

The results of β‐diversity analyses indicated that, across all four study lake systems and both hydrological seasons, turnover (Figure 5) mainly dominated the taxonomic β‐diversity of both phytoplankton and zooplankton, whereas nestedness (Figure 6) primarily dominated functional β‐diversity. The only exceptions occurred during the dry season for phytoplankton in HYH (*β*_sim_ = 54.70%, *β*_sne_ = 45.30%) and zooplankton in CZH (*β*_sim_ = 53.71%, *β*_sne_ = 46.29%). Higher values were generally observed in the dry season for both functional and taxonomic β‐diversity, although this pattern varied among lake systems and plankton groups. The values of total β‐diversity (*β*_sor_), turnover (*β*_sim_), and nestedness (*β*_sne_) were significantly lower in the functional dimension than in the taxonomic dimension.

### 3.3 Ecological Processes and Driving Mechanisms of Plankton Community Assembly

The neutral model did not fit the plankton community profile, with low migration rates or poor model fit (*R* ^2^ < 0) (Figure S3). This indicates that stochastic processes were not essential in governing the ecological processes of plankton communities, and the assembly of plankton communities in the sampled lakes was more likely regulated by deterministic processes (niche differentiation).

**FIGURE 7:**
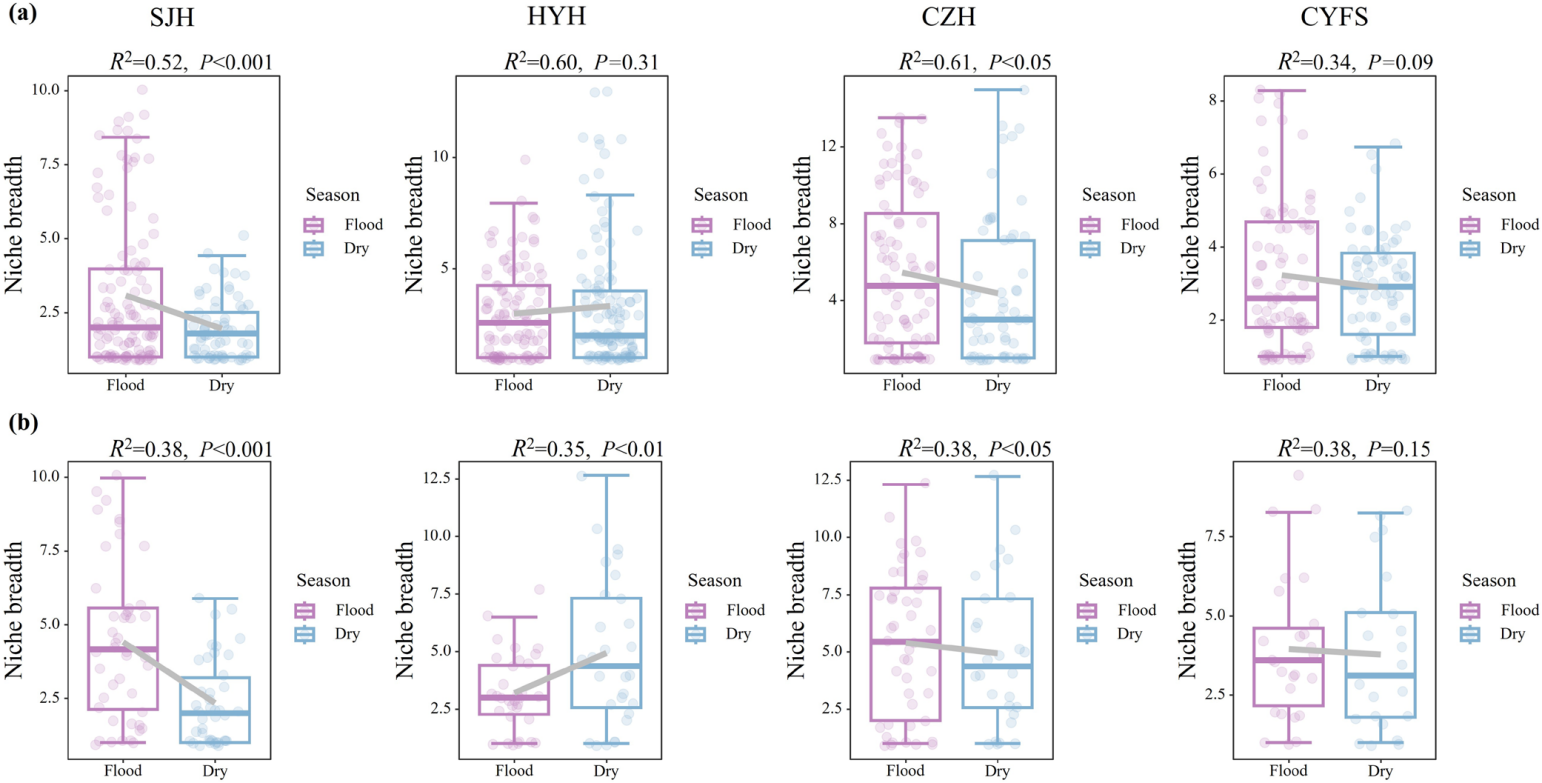
Spatiotemporal variation in plankton community niche breadth. (a) Phytoplankton. (b) Zooplankton.

To further examine differences in environmental adaptation and seasonal dynamics between phytoplankton and zooplankton, niche breadth was quantified at each sampling site (Figure 7). The GLM results showed seasonal differences in niche breadth among lakes. From the flood season to the dry season, the mean niche breadth of both phytoplankton and zooplankton decreased in SJH, CZH, and CYFS, but increased in HYH. For phytoplankton, the decrease was significant in SJH (*p* < 0.001) and CZH (*p* < 0.05), but not significant in HYH (*p* = 0.31) or CYFS (*p* = 0.09). For zooplankton, niche breadth decreased significantly in SJH (*p* < 0.001) and CZH (*p* < 0.05), increased significantly in HYH (*p* < 0.01), and showed no significant difference in CYFS (*p* = 0.15).

### 3.4 Biogeographical Analysis of the Plankton Communities

**FIGURE 8:**
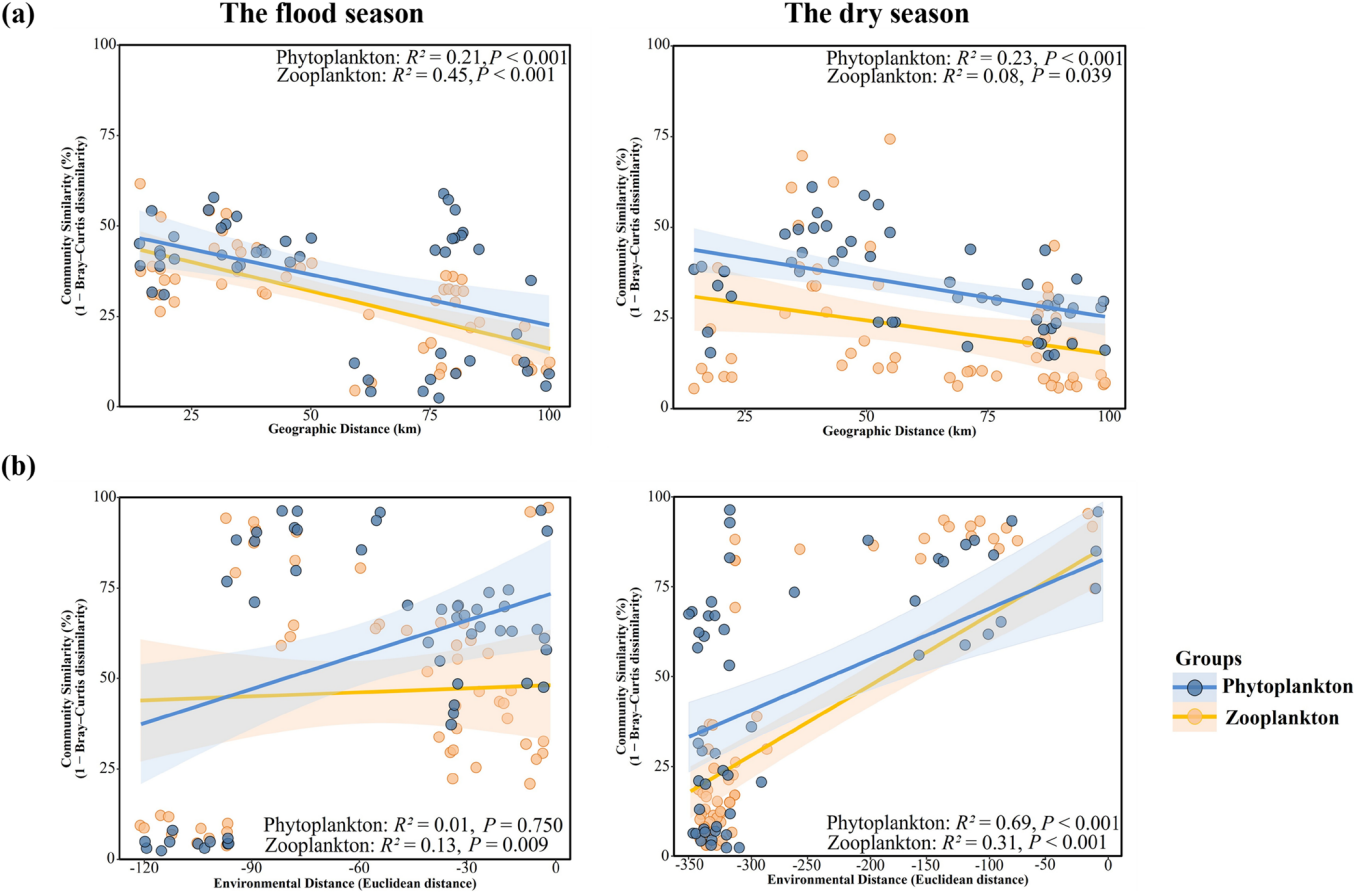
Distance–decay relationships of plankton communities. (a) Relationship between geographical distance and community similarity (calculated as 1 − Bray–Curtis dissimilarity) of phytoplankton and zooplankton communities; (b) Relationship between environmental distance (based on Euclidean distance of environmental variables) and community similarity (1 − Bray–Curtis dissimilarity) of phytoplankton and zooplankton communities.

Among all sampling sites, community similarity (1 − Bray–Curtis dissimilarity) of both phytoplankton and zooplankton communities decreased significantly with increasing geographical distance (*p* < 0.05). The geographical distance–decay relationship was stronger for zooplankton than for phytoplankton during the flood season (Phytoplankton: *R* ^2^ = 0.21; Zooplankton: *R* ^2^ = 0.45), whereas it was stronger for phytoplankton than for zooplankton during the dry season (Phytoplankton: *R* ^2^ = 0.23; Zooplankton: *R* ^2^ = 0.08) (Figure 8a). In addition, community similarity decreased significantly with increasing environmental distance for zooplankton during the flood season (*R* ^2^ = 0.13, *p* = 0.009) and for both phytoplankton (*R* ^2^ = 0.69, *p* < 0.001) and zooplankton (*R* ^2^ = 0.31, *p* < 0.001) during the dry season, whereas no significant relationship was detected for phytoplankton during the flood season (*R* ^2^ = 0.01, *p* = 0.750) (Figure 8b). During the dry season, the environmental distance–decay relationship was stronger for phytoplankton than for zooplankton, whereas during the flood season, only zooplankton showed a significant but weak relationship.

### 3.5 Relationships Between Plankton Communities and Environmental Factors

**FIGURE 9:**
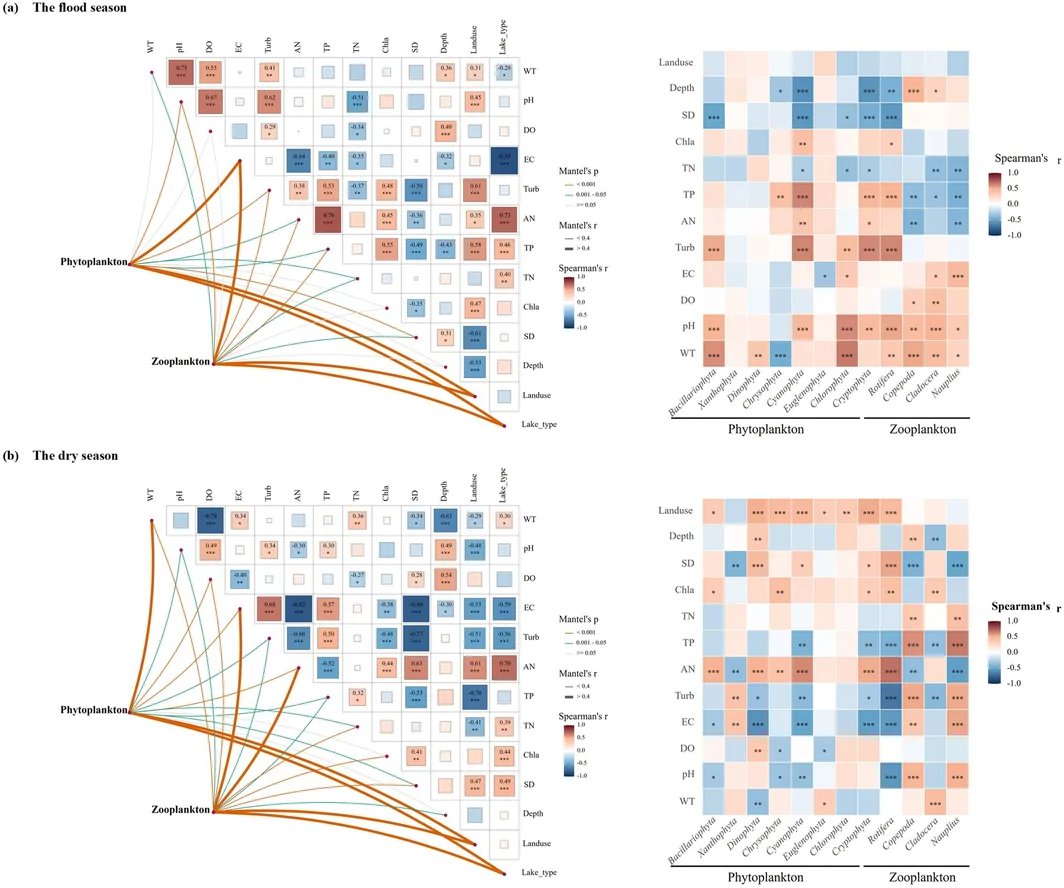
Environmental variables affecting the spatiotemporal pattern of plankton communities. (a) Environmental drivers of plankton communities and correlation of plankton phylum to environmental variables during the flood season; (b) Environmental drivers of plankton communities and correlation of plankton phylum to environmental variables during the dry season. Water temperature (WT), dissolved oxygen (DO), electrical conductivity (EC), turbidity (Turb), Secchi depth (SD), ammonia (AN), total nitrogen (TN), total phosphorus (TP), and water depth (WD). *p < 0.05, **p < 0.01, and ***p < 0.001. In the left panels, the upper triangular matrix represents Spearman correlations among environmental variables. The connecting lines represent Mantel correlations between phytoplankton or zooplankton community composition and individual environmental variables. Line color indicates the Mantel test *p*‐value, whereas line width indicates the magnitude of Mantel's *r*.

To explore the impacts of various factors on plankton groups in different seasons, we ran Mantel tests on environmental factors and plankton communities (phytoplankton and zooplankton) (Figure 9). In the flood season, electrical conductivity, land‐use types, and lake types correlated highly with plankton community composition (*R* > 0.4, *p* < 0.001) (Figure 9a). In the dry season, four environmental variables (electrical conductivity, ammonia nitrogen, land‐use types, and lake types) indicated a strong link with zooplankton communities (*R* > 0.4, *p* < 0.001). The main environmental factors affecting phytoplankton composition (*R* > 0.4, *p* < 0.001) were water temperature, land‐use types, and lake types (Figure 9b).

Further, Spearman's rank correlation analysis was used to examine the specific effects of environmental factors on plankton community structures (Figure 9). During the flood season, environmental factors mainly affected the relative abundance of Bacillariophyta, Cyanobacteria, Chlorophyta, and Cryptophyta in phytoplankton. Cyanobacteria and Cryptophyta were negatively correlated with water depth and Secchi depth (*p* < 0.001), and positively correlated with TP, turbidity, and pH (*p* < 0.01). Bacillariophyta and Chlorophyta were significantly negatively correlated with SD (*p* < 0.05), but significantly positively correlated with turbidity, water temperature, and pH (*p* < 0.05). Among zooplankton, SD negatively affected Rotifera, whereas turbidity had a positive effect (*p* < 0.001). Cladocera and Copepoda were positively correlated with depth, DO, pH, and WT and negatively correlated with TP; Copepoda was also negatively correlated with AN, whereas Cladocera was negatively correlated with TN.

In contrast, during the dry season, the relationships between zooplankton/phytoplankton communities and environmental factors displayed a different pattern compared to the flood season. In phytoplankton communities, the influence of environmental factors on Chlorophyta and Bacillariophyta weakened, whereas their impact on Chrysophyta, Dinophyta, and Xanthophyta intensified. Ammonia nitrogen and land‐use demonstrated notable correlations with most phytoplankton groups (*p* < 0.05). In zooplankton communities, land‐use was significantly positively correlated with Rotifera (*p* < 0.001). Zooplankton groups were also significantly correlated with SD, Chla, TN, TP, AN, turbidity, EC, pH, WT, and water depth.

**FIGURE 10:**
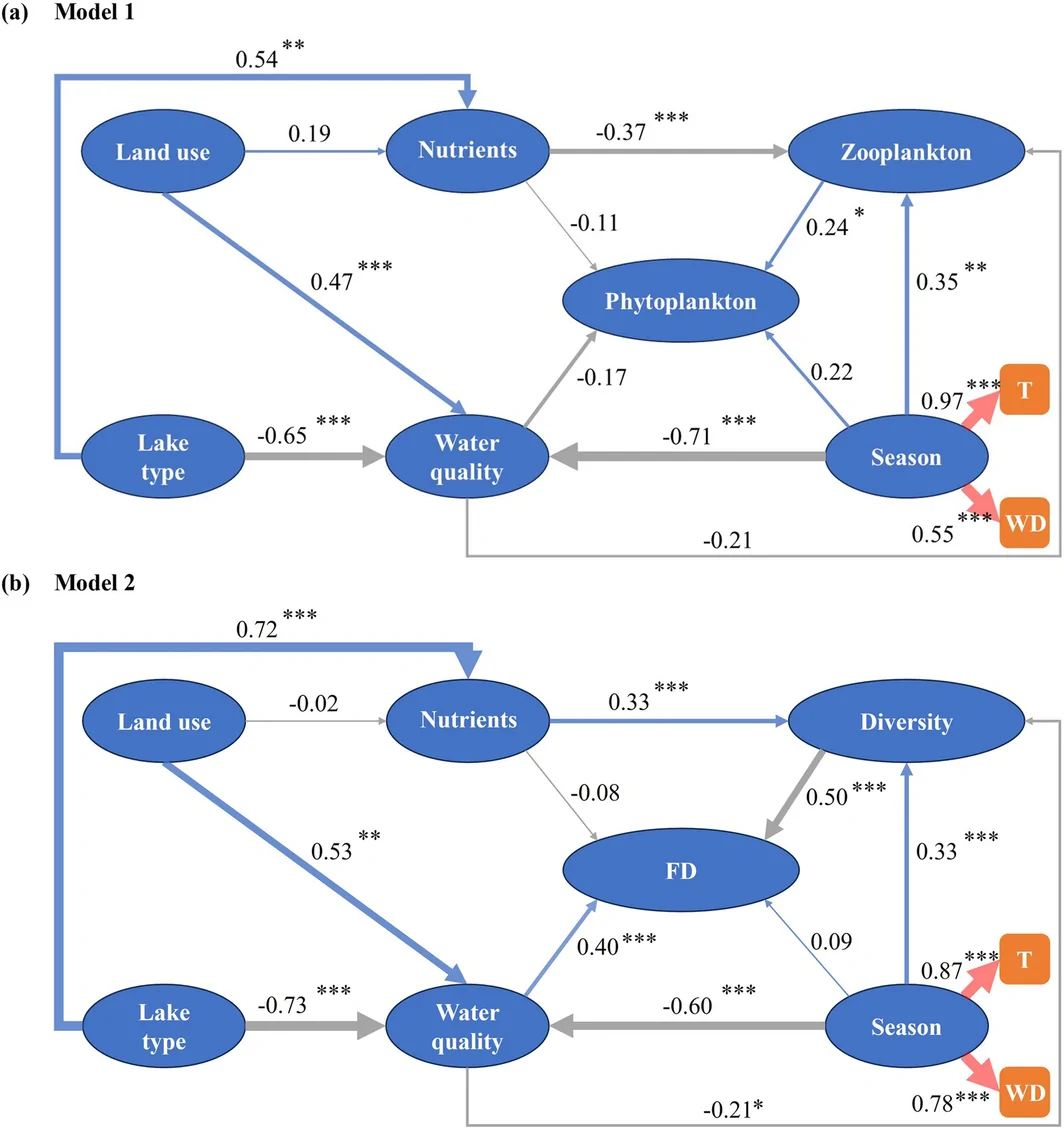
Effects of global change on plankton density and biodiversity (*n* = 105). (a) Partial least squares‐structural equation modeling (PLS‐SEM) for plankton diversity; (a) Partial least squares‐structural equation modeling (PLS‐SEM) for biodiversity. Functional diverasity (FD), water temperature (T), water depth (WD). *p < 0.05, **p < 0.01, and ***p < 0.001.

PLS‐SEM was used to assess the direct and indirect effects of lake characteristics, land use, season, water quality, and nutrients on plankton density/biodiversity. PLS‐SEM path analysis revealed that land‐use types (agriculture and urbanization) drive phytoplankton biomass changes by altering nutrient inputs (TN, TP, AN) and water quality parameters (DO, Turb) (Figure 10). Model 1 (GOF = 0.428) and Model 2 (GOF = 0.487) showed moderate explanatory power.

In Model 1, lake types negatively altered the water quality parameters (*β* = −0.65, *p* < 0.001) while significantly improving its nutrients (*β* = 0.54, *p* < 0.01). Land‐use types positively affected water quality (*β* = 0.47, *p* < 0.001) and weakly correlated with nutrients (*β* = 0.19, *p* > 0.05). Nutrients shrank the zooplankton density appreciably (*β* = −0.37, *p* < 0.001) but only reduced the phytoplankton density by a small amount (*β* = −0.11, *p* > 0.05). Season caused the zooplankton density (*β* = 0.35, *p* < 0.01) to swell. This indirectly brought about a surge in the phytoplankton density (*β* = 0.24, *p* < 0.05). However, season and water quality were significantly negatively correlated (*β* = −0.71, *p* < 0.001).

Model 2 results showed that land use markedly improved water quality (*β* = 0.53, *p* < 0.01). On the other hand, lake types diminished water quality (*β* = −0.73, *p* < 0.001), but had a notable positive effect on nutrients (*β* = 0.72, *p* < 0.001). Biodiversity was positively affected by nutrients (*β* = 0.33, *p* < 0.001) and season (*β* = 0.33, *p* < 0.001), and negatively affected by water quality (*β* = −0.21, *p* < 0.05). Water quality had a significant positive effect on functional diversity (*β* = 0.40, *p* < 0.001), and biodiversity and functional diversity were significantly positively correlated (*β* = 0.50, *p* < 0.001).

## 4 Discussion

### 4.1 The Temporal Dynamics of Plankton Community Characteristics

Understanding the temporal dynamics of plankton is essential for interpreting how plankton communities respond to seasonal environmental variation in floodplain lakes. Consistent with our hypothesis, we observed pronounced temporal dynamics in plankton characteristics across the four floodplain lake types, with higher density and α‐diversity but lower β‐diversity during the flood season. These patterns indicate a transition from spatially heterogeneous communities in winter to more homogenized assemblages during summer flooding.

In the study lakes, the flood season generally occurs from May to August, coinciding with summer, when hydrological and environmental conditions undergo marked changes. Our observations showed that plankton density increased rapidly during this period and reached annual maxima, providing direct support for the observed seasonal peak in abundance. Similar seasonal patterns have been reported in subtropical aquatic ecosystems (Portner and Farrell 2008; Roncalli et al. 2022). Concurrently, rising water levels and expanded water surface area were associated with changes in habitat availability for plankton. Seasonal runoff was accompanied by increased concentrations of nitrogen and phosphorus, indicating enhanced external nutrient input during flood season (Dembowska 2015; Wang, Gao, et al. 2021; Wang, Wang, et al. 2021; Happe et al. 2025; Yang and Zhao 2024). Although flooding may reduce plankton density through dilution, this effect in the studied lakes was likely partly offset by warmer temperatures and increased nutrient availability, which promoted rapid plankton growth. Together, these conditions were closely associated with increased plankton abundance and species richness, as reflected by higher α‐diversity indices in the flood season.

Beyond taxonomic richness, functional α‐diversity also increased during the flood season. This pattern likely reflects the coexistence of a broader range of functional strategies under favorable environmental conditions, rather than direct evidence of intensified biotic interactions (Wellard Kelly et al. 2021). Meanwhile, enhanced hydrological connectivity and water mixing during the flood season were associated with reduced environmental heterogeneity among lakes (Peršić and Horvatić 2011; Weigelhofer et al. 2011). As a result, plankton community composition became more similar across sites, leading to lower taxonomic and functional β‐diversity during the flood season, in line with the general patterns observed in plankton of other floodplain systems (Amaral et al. 2024; Li et al. 2025).

Seasonal shifts were also evident in the dominance patterns of major plankton groups. Phytoplankton communities were primarily composed of Cyanobacteria, Chlorophyta, Bacillariophyta, and Cryptophyta, whereas Rotifera dominated zooplankton assemblages throughout the year, consistent with previous studies in floodplain lakes (Zhang, Xu, et al. 2018; Zhang et al. 2019; Wang, Gao, et al. 2021; Wang, Wang, et al. 2021). Cyanobacteria were particularly dominant during the flood season in most lakes, likely favored by higher water temperatures, longer daylight duration, and elevated nutrient availability (Ai et al. 2024; Wang et al. 2025; Yang et al. 2023). In contrast, their dominance weakened during the dry season. Lower temperatures and reduced light conditions during winter coincided with increased contributions from Bacillariophyta, Chlorophyta, and Cryptophyta, especially taxa such as *K. caudata* and *C. ovata* , which are known to tolerate low‐light environments (Ding et al. 2023).

Rotifera consistently dominated zooplankton communities across seasons, a pattern commonly observed in subtropical lakes, reservoirs, and rivers (Sharma 2005; Wang et al. 2024). Their small body size, rapid reproduction, high feeding efficiency, and broad ecological tolerance enable them to thrive under warm, nutrient‐rich conditions in summer and to persist during unfavorable winter conditions through resting egg production (Lair 2006). Consequently, rotifers play a central role in structuring zooplankton communities in floodplain lakes.

At the level of β‐diversity partitioning, taxonomic β‐diversity was largely driven by species turnover, whereas functional β‐diversity was dominated by nestedness. Similar patterns have been reported across a wide range of aquatic ecosystems (Schartau et al. 2022; Zhang, Qu, et al. 2023; Zhang, Li, et al. 2023). In the study region, floodplain lakes are distributed along the Yangtze River, and hydrological isolation caused by sluice gates likely limits plankton dispersal among lakes, thereby enhancing species turnover. Meanwhile, differences in lake type and surrounding land use create environmental gradients that filter species composition while maintaining core functional traits across systems, resulting in pronounced functional nestedness (Martínez‐Leiva et al. 2025). The lower functional β‐diversity relative to taxonomic β‐diversity further suggests high functional redundancy within plankton communities (Jia et al. 2025).

### 4.2 The Assembly Processes of Plankton Communities in Floodplain Lakes

Understanding the relative importance of deterministic and stochastic processes in community assembly is a central topic in ecology, particularly in spatially fragmented aquatic systems such as floodplain lakes. In this study, multiple analytical results consistently indicated that deterministic processes played a dominant role in structuring plankton communities across floodplain lakes, while stochastic processes were detectable but secondary.

The lakes in this study are sluice‐regulated, with markedly constrained hydrological connectivity that directly limits the dispersal of plankton (especially phytoplankton) and consequently impedes community homogenization across lakes. Meanwhile, reduced water exchange further amplifies the divergence of local environmental conditions, as evidenced by the differences in physicochemical parameters among lake types. This environmental heterogeneity selects for plankton taxa adapted to their respective aquatic conditions via deterministic mechanisms, exerting a strong environmental filtering effect (Hu et al. 2019; Masclaux et al. 2015; Rao et al. 2018). Consistent with the observed associations between plankton community structure and environmental variables, our results indicate that the compositional divergence among lake types arises from the combined effects of dispersal limitation and environmental filtering.

Environmental filtering acts across both spatial and temporal scales: beyond the spatial heterogeneity among lakes, predictable seasonal hydrological fluctuations in these subtropical floodplain lakes provide a stable temporal framework for plankton community succession, driving cyclical shifts in dominant taxa and community structure (Li et al. 2013; Song et al. 2010; Tan et al. 2009). This filtering process not only constrained species occurrence but also shaped the functional trait composition of plankton communities. During the flood season, higher water levels and expanded habitat space, and relatively high water temperature and nutrient availability were associated with increased taxonomic and functional α‐diversity. This pattern likely reflects the coexistence of a wider range of functional strategies under favorable environmental conditions, rather than direct evidence of intensified biotic interactions. Functional differentiation among plankton taxa may facilitate niche partitioning within communities. For example, *Microcystis* sp. can regulate its buoyancy through gas vesicles and maintain access to light near the water surface, whereas many *Navicula* taxa are benthic or tychoplanktonic and may exploit low‐light conditions and sediment‐associated nutrients (Jöhnk et al. 2008; Wu et al. 2023). Nitrogen‐fixing cyanobacteria such as *Anabaena* sp. can use atmospheric N_2_, while non‐nitrogen‐fixing algae rely on dissolved inorganic nitrogen in the water. These contrasting resource‐use strategies may facilitate coexistence in nutrient‐rich lakes (Liu et al. 2025; Wei et al. 2023).

Environmental filtering provides an important framework for community assembly and represents a key deterministic process. In this study, our results indicate that lake‐specific environmental conditions shaped by hydrological isolation and predictable seasonal dynamics played a central role in structuring plankton communities. Stochastic processes such as dispersal limitation and ecological drift likely operated concurrently but appeared to exert a secondary influence under the environmental context examined here (Jin et al. 2022; Sauterey et al. 2017).

### 4.3 Biogeographical Patterns of Plankton Communities

The biogeographical patterns of plankton communities differed markedly between phytoplankton and zooplankton across the studied floodplain lakes. NMDS ordination revealed that zooplankton communities exhibited greater spatial variation but weaker temporal differentiation than phytoplankton, suggesting contrasting sensitivities to spatial heterogeneity and seasonal environmental change between the two groups.

The pronounced spatial differentiation of zooplankton communities may also be associated with variability in local habitat characteristics among lakes, including bathymetry, vegetation structure, and sediment composition. Such habitat heterogeneity may influence zooplankton survival and reproductive success, thereby contributing to spatially structured community patterns (Ji et al. 2025; Zhang et al. 2025). In contrast, the relatively weak temporal response of zooplankton communities is constrained by cascading trophic interactions within plankton food webs (Elias and Ursula 2020).

Hydrological connectivity emerged as a key factor shaping similarities among plankton communities across lake types. Despite being classified as different lake types, SJH and CZH harbored highly similar plankton assemblages. During the sampling period, water diversion in CZH connected its water source to the Yangtze River, resulting in physicochemical conditions comparable to those of the river and, consequently, plankton communities resembling those in the naturally connected SJH. This convergence underscores the strong homogenizing effect of hydrological connection on biogeographical patterns in floodplain lakes. By contrast, the reservoir‐type lake CYFS exhibited plankton community characteristics typical of subtropical reservoirs (Ni et al. 2023; Xu et al. 2025).

HYH represented a striking exception within the study lakes, exhibiting a distinct plankton community structure relative to the other lakes. Phytoplankton communities in HYH were characterized by a higher contribution of Chlorophyta, while zooplankton assemblages showed relatively even contributions among major groups rather than dominance by Rotifera. This distinctive pattern coincided with the complex hydrological configuration of HYH, which comprises three interconnected lakes with contrasting habitat features and frequent internal water exchange. High habitat heterogeneity and connectivity may suppress the dominance of filamentous cyanobacteria and favor Chlorophyta, as observed in systems with enhanced water exchange (Phlips et al. 2008). In addition, extensive aquatic vegetation and dynamic hydrological conditions likely provide refugia and food resources for Cladocera and Copepoda, contributing to their elevated relative abundances (Meyer et al. 2018; Yousef et al. 2024). The higher contribution of nestedness to phytoplankton functional β‐diversity in HYH during the dry season may further reflect strong environmental gradients generated by frequent water exchange.

Distance–decay relationships provided complementary insights into the processes governing plankton biogeography. Both geographic and environmental distances were significantly correlated with plankton community dissimilarity, indicating that dispersal limitation and environmental filtering jointly shape community composition (Hou et al. 2025; Zhang, Xu, et al. 2018). However, the relative importance of these processes varied between plankton groups and seasons.

During the flood season, zooplankton communities exhibited stronger distance–decay relationships with both geographic and environmental distance than phytoplankton, suggesting greater sensitivity to spatial separation and habitat differentiation. In contrast, during the dry season, phytoplankton community composition showed a particularly strong association with environmental distance (*R* ^2^ = 0.69), exceeding that with geographic distance (*R* ^2^ = 0.23), highlighting the dominant role of environmental filtering. Zooplankton communities also displayed a significant relationship with environmental distance in the dry season (*R* ^2^ = 0.31), despite weak geographic distance decay, indicating enhanced environmental control under low‐water conditions.

These contrasting seasonal patterns likely arise from differences in life‐history strategies and ecological traits between phytoplankton and zooplankton. Phytoplankton generally have short generation times and are readily dispersed by water flow, allowing rapid responses to resource availability and hydrological connectivity. In contrast, zooplankton have relatively longer generation times, greater mobility, and stronger dependence on food availability and predator–prey interactions, making their distributions more sensitive to local environmental conditions. During the flood season, high hydrological connectivity and abundant resources may weaken the influence of environmental gradients on phytoplankton communities, resulting in a negligible relationship with environmental distance (*R* ^2^ = 0.01, *p* = 0.75). Under such conditions, phytoplankton communities are likely structured by rapid growth and widespread dispersal, consistent with r‐selected strategies (Song et al. 2010). Conversely, during the dry season, reduced water levels and lower functional redundancy may increase the sensitivity of plankton communities to environmental variation, thereby strengthening the role of environmental filtering in shaping community composition.

### 4.4 Driving Factors Influencing Plankton Communities and Coupling Effects of Environmental Variables in Floodplain Lakes

Consistent with our expectation that hydrological seasonality shapes plankton communities, distance–decay relationships (DDRs) and Spearman rank correlations revealed that seasonality was the dominant driver of community dynamics in floodplain lakes. Seasonal fluctuations in water level and water temperature structured plankton responses by modifying key physical and chemical conditions. During the flood season, Secchi disk depth (SD), turbidity, and pH were the primary water‐quality variables influencing community composition. Reduced water transparency and elevated turbidity altered the underwater light environment. The small body size, rapid reproductive rates, flexible feeding strategies, and broad environmental tolerance of rotifers may have contributed to their dominance under these fluctuating environmental conditions (Hasnain and Arnott 2019; Li et al. 2022). Lower light availability also restricted phytoplankton photosynthesis while promoting low‐light‐adapted groups such as Cryptophyta (Ding et al. 2023; Yang et al. 2023). Although pH remained within the neutral to slightly alkaline range in both seasons, its relatively higher values during the flood season may have favored algal metabolic activity and contributed, together with other environmental factors, to seasonal variation in phytoplankton communities (Heneash et al. 2015; Lin et al. 2025).

In contrast, plankton communities during the dry season exhibited broader responses to multiple water‐quality parameters. The relative dominance of Bacillariophyta, Cyanobacteria, Chlorophyta, Cryptophyta, and rotifers declined, whereas the proportions of Chrysophyta, Dinophyta, Xanthophyta, Cladocera, and Copepoda increased, indicating enhanced functional differentiation under greater environmental heterogeneity. Winter TP concentrations in the Yangtze River source region were generally low, likely due to reduced precipitation and surface runoff, which limited external phosphorus inputs, together with suppressed internal phosphorus release under low‐temperature conditions. Structural equation modeling (SEM) further indicated that seasonal effects on plankton diversity were largely mediated through changes in water quality. Elevated water temperatures were associated with reduced dissolved oxygen concentrations, which indirectly influenced both taxonomic and functional diversity (Cervania and Hamme 2024). Functional diversity was also affected indirectly via changes in taxonomic diversity, highlighting a hierarchical linkage between these two dimensions.

Mantel test results indicated that land use and lake type remained significantly associated with plankton community structure across seasons. Land use, as a spatial manifestation of anthropogenic activities, influenced plankton communities primarily through its effects on water quality and nutrient inputs. Agricultural and urban land uses represented major nutrient sources in subtropical floodplain lakes. Fertilizer application in agricultural areas increased nitrogen and phosphorus loading, stimulating phytoplankton growth and altering community structure and functional attributes (Lv et al. 2024; Zhang, Cai, et al. 2024; Zhang, Liu, and Chen 2024). Urban areas contributed organic matter and heavy metals via domestic wastewater discharge, modifying water physicochemical properties and nutrient bioavailability and thereby affecting plankton communities (Godziejewska et al. 2024).

During the flood season, intensified precipitation enhanced surface runoff, leading to fluctuations in electrical conductivity and ammonia nitrogen concentrations that promoted plankton growth and metabolic activity (Li et al. 2021; Song et al. 2024). Lake type further modulated these processes by regulating hydrological connectivity, water exchange, and mixing intensity. Lakes with stronger river–lake connectivity exhibited higher water renewal rates and more homogeneous oxygen and nutrient distributions, conditions favorable for plankton growth.

## 5 Conclusions

This study conducted an in depth analysis of the temporal dynamics, community assembly, biogeographic patterns, and environmental responses of plankton communities in subtropical floodplain lakes. The results showed that the plankton communities were mainly composed of Cyanobacteria, Chlorophyta, Bacillariophyta, Cryptophyta, and Rotifera. The density and alpha diversity of plankton were higher during the flood season than during the dry season, whereas beta diversity was higher during the dry season. Taxonomic beta diversity was dominated by turnover, while functional beta diversity was dominated by nestedness. Environmental filtering drove the community assembly of plankton, which was mainly deterministic, and such deterministic community dynamics further led to niche differentiation. Compared with phytoplankton, zooplankton exhibited greater spatial variation but lower temporal variation. Distance–decay relationships indicated that both geographic distance and environmental distance influenced community structure, and their relative importance varied with season and taxonomic group. Seasonal variations are key drivers of plankton dynamics, with distinct water quality factors influencing plankton responses across seasons. Land use and lake type indirectly affect plankton characteristics through coupling effects with water quality and nutrients. This study enhances our understanding of plankton community characteristics in subtropical floodplain lakes and provides a scientific basis for the ecological management and protection of floodplain lake ecosystems.

## Author Contributions

**Lingli Jiang:** investigation (equal), methodology (lead), visualization (lead), writing – original draft (lead). **Zhongze Zhou:** funding acquisition (lead), investigation (equal). **Bohan Zhou:** investigation (equal). **Yaqiang Yuan:** investigation (equal). **Su Mei:** investigation (equal). **Yuqian Liu:** investigation (equal). **Yutao Wang:** writing – review and editing (equal).

## Funding

This research was supported by Joint Research Project for the Yangtze River Conservation (Phase I), China (Nos. 2019‐LHYJ‐01‐0212, 2019‐LHYJ‐01‐0212‐17).

## Conflicts of Interest

The authors declare no conflicts of interest.

## Supporting information


**Figure S1:** Maps of sampling sites for study lakes in different seasons.
**Figure S2:** Heat map analysis based on the cell density of the plankton dominant species.
**Figure S3:** The neutral community model of plankton community.

**Table S1:** The list of zooplankton characteristics.
**Table S2:** The list of phytoplankton characteristics.
**Table S3:** Environmental factors characteristics of the study area (mean value ± SD).
**Table S4:** The α‐diversity of plankton (mean value ± SD). *d*, Margalef's index; FDis, functional dispersion index; FEve, functional evenness index; Fric, functional richness index; *H*′, Shannon–Wiener's index; *J*′, Pielou's evenness index.
**Table S5:** Density of different plankton taxa.

## Data Availability

The data that supports the findings of this study are available in the Supporting Information of this article.
