# Supplementary material for: What Drives the Changing Characteristics of Plankton in Floodplain Lakes: Season, Environmental Factors, or Lake Characteristics?
Source: Ecol Evol. 2026 Sep 9;16(9):e74277. doi: 10.1002/ece3.74277 (PMC13557922; doi:10.1002/ece3.74277)
Supplement: Supplementary file 1 [file ECE3-16-e74277-s001.docx]

**Fig. S1. Maps of sampling sites for study lakes in different seasons.**

**Fig. S2.** **Heat map analysis based on the cell density of the plankton dominant species.** (a) Phytoplankton; (b) Zooplankton. Note: The value of the cell density was logarithmically transformed to more clearly show the proportions of each species of phytoplankton. a value of zero means that the species was not identified.

**Fig.S3. The neutral community model of plankton community.** (a) Phytoplankton during the flood season; (b) Phytoplankton during the dry season; (c) Zooplankton during the flood season; (b) Zooplankton during the dry season.
